# Three novel neoplasms in Nancy Ma’s owl monkeys (*Aotus nancymaae*)

**DOI:** 10.1177/03009858241300549

**Published:** 2024-12-18

**Authors:** Rebecca L Bacon, Carolyn L Hodo, Stephanie J Buchl, Martha E Hensel

**Affiliations:** 1 Texas A&M University, College Station, TX; 2 Duke University, Durham, NC; 3 The University of Texas MD Anderson Cancer Center, Bastrop, TX

**Keywords:** *Aotus spp.*, cervical carcinoma, epithelioid trophoblastic tumor, mesothelioma, neoplasia, nephroblastoma, owl monkey

## Abstract

Neoplasms are only sporadically reported in New World primates and rarely in owl monkeys (*Aotus spp.*), specifically. Previous reports of neoplasms in owl monkeys are primarily restricted to lymphoma induced by *Herpesvirus saimiri* infection, although other tumors in the central nervous, genitourinary, gastrointestinal, and endocrine systems have been sporadically reported. Herein, we describe 3 previously unreported neoplasms in owl monkeys (*Aotus nancymaae*) including a pericardial mesothelioma in a 6-year-old male, a nephroblastoma in a 2-year-old male, and a cervical neoplasm with characteristics of an epithelioid trophoblastic tumor in a 4-year-old female, all occurring in the same closed breeding colony at a research facility in central Texas. Reporting of spontaneously occurring neoplasms in research colony populations is important for identifying potential animal models of human diseases and for improving colony management and species health.

Neoplasms are sporadically reported in New World nonhuman primate (NHP) species used in biomedical research including marmosets (*Callithrix sp.*), squirrel monkeys (*Saimiri sp.*), tamarins (*Sanguinus sp.*), capuchin monkeys (*Cebus sp.*), and owl monkeys (*Aotus sp.*).^17^ Reports of spontaneous neoplasia in owl monkeys are infrequent. However, owl monkeys may develop lymphoma when experimentally or spontaneously infected with *Herpesvirus saimiri*.^1,2,17^ Single case reports have documented spontaneous neoplasms outside of the hematopoietic system including a seminoma, an oligodendroglioma, a gastric leiomyoma, 2 renal papillary carcinomas, a generally described “benign adrenal tumor,” and a pancreatic endocrine tumor.^1,2,4,11,12,15,17,18^ Infrequent reports of neoplasia in *Aotus sp.*, particularly when compared with reports in old world primates, may be due in part to the low numbers of this species used in biomedical research and the young age of the research population. However, as the research breeding population ages, neoplasia is likely to be diagnosed more frequently. Herein, we describe previously unreported cases of spontaneous neoplasia of the heart and pericardial sac, kidney, and cervix in 3 adult owl monkeys.

Adult owl monkey (>1 year of age) necropsy records from a 13-year period (2008 to 2021) at the Michale E. Keeling Center for Comparative Medicine and Research at The University of Texas MD Anderson Cancer Center were reviewed. The Owl Monkey Breeding and Research Resource was formed in 2001 and moved to the Michale E. Keeling Center for Comparative Medicine and Research in 2008. Closed breeding colonies of 4 species are housed within the Owl Monkey Breeding and Research Resource: *Aotus nancymaae*, *A. azarae*, *A. vociferans*, and *A. griseimembra*, along with a small number of hybrid individuals that are not used for breeding. Of the 225 cases reviewed, the majority of neoplasms involved the digestive system, which have been previously reported.^14^ During this time period, spontaneous neoplasia was identified in 16 of 225 cases. Of the identified cases of spontaneous neoplasia, 3 outside of the digestive system were identified. The Michale E. Keeling Center for Comparative Medicine and Research is an Association for Assessment and Accreditation of Laboratory Animal Care accredited facility. Animals are cared for in accordance with the United States Department of Agriculture (USDA) Animal Welfare Act and regulations and the *Guide for the Care and Use of Laboratory Animals* and established Institutional Animal Care and Use Committee policies.^23^ All cases were experimentally naïve colony animals and underwent a complete necropsy according to institutional procedures. Tissues were fixed in 10% neutral-buffered formalin, and samples for histology were routinely processed and embedded, cut at 4 µm, stained with hematoxylin and eosin, and examined by light microscopy. Case 2 was initially diagnosed based on a surgical biopsy, although the animal was euthanized and necropsied later. Archived histology from each case was reviewed to confirm the diagnosis by 2 board-certified pathologists. In case 1, immunohistochemistry was performed to aid in diagnosis using anti-pancytokeratin AE1/AE3 (1:100, Biocare Medical) (epithelial) and anti-vimentin (SP20; 1:100, Biocare Medical) (mesenchymal) antibodies. In case 3, immunohistochemistry with several antibodies was attempted but unsuccessful in this species. Details of the antibodies and methods used are provided in Supplemental Table S1.

Case 1 was a 6-year-old male *A. nancymaae* with no record of previous clinical disease. The animal appeared lethargic during a routine weight measurement, then became dyspneic and died acutely. Necropsy revealed a mild serosanguineous peritoneal effusion, and the thoracic cavity contained 30 mL of serosanguineous fluid. The upper lung lobes were atelectatic secondary to the effusion, and the lower left lung lobe had a 5-mm diameter, flat, tan mass firmly adhered to the pleural surface. Both the serosal and visceral surfaces of the pericardial sac and the epicardial surface of the heart were expanded by numerous 3 to 7 mm diameter, raised, tan nodules (Fig. 1a). Histology of the pericardial sac and heart (Fig. 1b) showed an infiltrative, unencapsulated, densely cellular neoplasm composed of pleomorphic polygonal cells arranged in dense islands and papillary projections (Fig. 1b, inset) supported by a moderate fibrovascular stroma. Neoplastic cells generally spread along the pleural, epicardial, and pericardial surfaces, but multifocally infiltrated into the underlying myocardium and invaded the vasculature. The neoplastic cells had distinct cell borders, a moderate-to-large amount of eosinophilic cytoplasm, and large oval to irregular nuclei with coarsely stippled chromatin and up to 3 large magenta nucleoli. Anisocytosis and anisokaryosis were marked with occasional bi- and multi-nucleation and bizarre mitoses. Foci of small lymphocytes were scattered throughout the neoplasm. Based on the gross and histologic features, a diffuse epithelioid mesothelioma was diagnosed.^24^ Neoplastic cells displayed diffuse cytoplasmic immunoreactivity for pancytokeratin and vimentin, which confirmed the diagnosis. Additional relevant findings included centrilobular hepatic necrosis (likely ischemia-related) and pulmonary thrombosis. Despite the presence of intravascular invasion of neoplastic cells, no distant metastases were noted.

**Figure 1.**
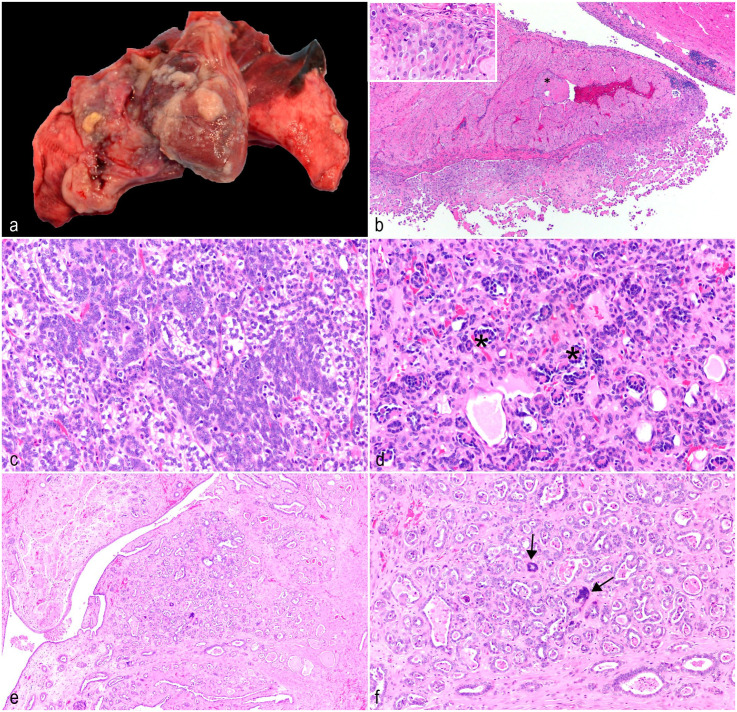
Neoplasms outside of the digestive tract in owl monkeys. (a) Intrathoracic, diffuse mesothelioma. Pericardium and pleura, *A. nancymaae*. The pericardium and pulmonary pleura have multifocal tan plaques adhered to the surfaces. (b) Intrathoracic, diffuse epithelioid mesothelioma. Heart, *A. nancymaae*. Neoplastic mesothelial cells infiltrate the myocardium and invade the vasculature. Vascular invasion is indicated by asterisk (*). Inset: Neoplastic cells have distinct cell borders, moderate-to-large amounts of eosinophilic cytoplasm, large oval to irregular nuclei with coarsely stippled chromatin, up to 3 large magenta nucleoli, occasional bi- and multi-nucleation, and bizarre mitoses. Hematoxylin and eosin (HE). (c) Nephroblastoma. Kidney, *A. nancymaae*. The predominant neoplastic population forms tubular structures lined by epithelial cells with scant basophilic cytoplasm on a spindle cell stroma similar to embryonal mesenchyme. HE. (d) Nephroblastoma. Kidney, *A. nancymaae*. Epithelial cells form invaginations on a fine fibrovascular stalk, resembling primitive glomeruli. Several examples are indicated by asterisks (*). HE. (e) Presumptive epithelioid trophoblastic tumor. Cervix, *A. nancymaae*. The neoplasm invades from the mucosa to the inner muscular layer with multifocally hyalinized stroma. Neoplastic cells appear to efface normal glandular structures. HE. (f) Presumptive epithelioid trophoblastic tumor. Cervix, *A. nancymaae*. Neoplastic cells are cuboidal to columnar with variably distinct cell borders, small amounts of eosinophilic cytoplasm, and central oval nuclei with coarsely stippled chromatin and 1 prominent basophilic nucleolus. There is a second population of large multinucleated syncytial cells that resemble syncytiotrophoblasts (arrows). HE.

Case 2 was a 2-year-old male *A. nancymaae* that presented with a palpable abdominal mass detected during a routine physical examination, but no other clinical signs were reported. A 5 × 3.2 × 3.4 cm, mottled, dark red mass replacing the left kidney was removed on exploratory laparotomy. Histologically, the mass effaced the kidney, with only a slim rim of normal renal tissue remaining. The neoplasm was unencapsulated, expansile, densely cellular, and composed of 3 distinct cell types: epithelial, stromal, and blastemal. The predominant population formed tubular structures which were either lined by epithelial cells with scant basophilic cytoplasm, or epithelial cells which were more plump, with clear cytoplasm. Both populations were supported by a loose spindle cell stroma similar to embryonal mesenchyme (Fig. 1c). Multifocally, neoplastic epithelial cells formed invaginations on a fine fibrovascular stalk, resembling primitive glomeruli (Fig. 1d). Finally, small areas of blastemal cells, characterized as streams of basophilic polygonal cells with a small amount of cytoplasm and indistinct cell borders, were scattered throughout the neoplasm. There were focally extensive areas of necrosis and hemorrhage, and a small portion of the residual renal cortex had a mixed, interstitial and intratubular immune cell infiltrate with fibrosis. The inflammatory infiltrate was primarily neutrophilic, with smaller numbers of mononuclear cells. Based on the characteristic features, the neoplasm was diagnosed as a nephroblastoma. The animal recovered well from the surgical procedure. However, less than 1 month later, overt renal disease developed as indicated by a blood urea nitrogen of over 140 mg/dl (reference range = 15-17 mg/dl) and potassium of less than 2 mEq/l (reference range = 3.3-4.8 mEq/l). The animal failed to improve with supportive therapy, and ultrasonographic examination of the remaining kidney revealed chronic nephropathy necessitating humane euthanasia. Necropsy and histology did not reveal metastasis of the neoplasm, but identified chronic diffuse glomerulonephropathy and mild oral candidiasis, presumably secondary to chronic debilitation.

Case 3 was a 4-year-old female *A. nancymaae* that underwent a cesarean section for dystocia. At the time of surgery, an enlarged bladder was noted with clinical concern for bladder atony. The animal did not urinate post-surgery, and the bladder could not be expressed via manual palpation. Based on the poor prognosis, humane euthanasia was performed. Necropsy revealed markedly dilated blood vessels in the pelvic canal and a thickened cervix. Examination of the mucosal aspect of the cervix revealed a multilobulated, tan, firm mass in the cervix, which likely constricted the urinary outflow tract. Histologically, the neoplasm was composed of invasive, irregular tubules on a moderate fibrovascular stroma which was multifocally hyalinized (Fig. 1e). The neoplastic tubules appeared to efface normal glandular structures (Fig. 1e, inset). Most of the neoplastic cells were cuboidal to columnar with variably distinct cell borders, small amounts of eosinophilic cytoplasm, and central oval nuclei with coarsely stippled chromatin and 1 prominent basophilic nucleolus. Multifocally throughout the neoplasm, there was a second population of large multinucleated syncytial cells with hyperchromatic nuclei, resembling syncytiotrophoblasts (Fig. 1f). Neoplastic tubules frequently contained karyorrhectic and proteinaceous debris. Epithelioid trophoblastic tumor (ETT), gestational choriocarcinoma, and cervical adenocarcinoma were considered as differential diagnoses for the neoplasm. Table 1, adapted from Quist et al,^20^ displays the primary diagnostic features of each. Based on the bland appearance of the cuboidal population, the presence of the multinucleated cells with hyperchromatic nuclei, and the replacement of normal glandular structures, a diagnosis of ETT was preferred, with cervical adenocarcinoma ruled out due to the presence of the multinucleated cells, which are not a feature of that tumor. The remainder of the post-mortem findings were considered incidental to the case.

**Table 1.** Diagnostic features of differential diagnoses for case 3.^a^

Epithelioid trophoblastic tumor	• Proliferation of medium-sized mononucleate trophoblastic cells
	• Occasional syncytiotrophoblastic cells
	• Moderate amount of finely granular eosinophilic or clear cytoplasm
	• Distinct cell borders
	• Low mitotic rate
	• Frequent necrosis and hyaline-like material
	• May colonize mucosal surfaces and/or glandular epithelium of cervix and endometrium
Gestational choriocarcinoma	• Primary population of infiltrative mononuclear villous cytotrophoblasts and intermediate trophoblasts
	• A surrounding ring of nucleated syncytiotrophoblasts
	• High mitotic rate
	• Frequent hemorrhage and necrosis
	• Frequent intravascular or intralymphatic invasion
Cervical adenocarcinoma	• Cuboidal to columnar cells
	• Distinct cell borders
	• Pale eosinophilic or foamy vacuolated cytoplasm
	• Variable nuclear atypia, may be severe
	• Scattered mitoses
	• Syncytiotrophoblastic cells not present

a Modified from Quist et al.^20^

This report expands our knowledge of neoplasms that occur in owl monkeys, including a pericardial mesothelioma (case 1), nephroblastoma (case 2), and a presumed ETT (case 3). The majority of the literature describing neoplasms in NHPs relies on case reports because the frequency of spontaneous neoplasia is estimated at only 5%.^17^ Of the cases reviewed for this study surveying MD Anderson Cancer Center’s colony of owl monkeys, 6% were identified with neoplasia, with just 3 occurring outside of the digestive tract.

Spontaneous mesotheliomas have been reported in the peritoneum of a Japanese macaque (*Macaca fuscata*) and the pericardium of a rhesus macaque (*Macaca mulatta*). The biological behavior of the tumors varied between these 2 cases. The peritoneal mesothelioma caused ascites and resulted in euthanasia, whereas the pericardial mesothelioma was found incidentally during a research study necropsy.^5,25^ Mesotheliomas are associated with asbestos exposure in humans, but a similar association with asbestos has not been linked to naturally occurring cases in veterinary species. These aforementioned macaque examples were both mature to geriatric adults, in contrast to the owl monkey presented here, which was a young adult.

Nephroblastomas have been reported in NHPs, including rhesus macaques (*M. mulatta*), cynomolgus macaques (*Macaca fascicularis*), baboons (*Papio sp.*), and a cotton-top tamarin.^3,8,15^ They are a common renal tumor of humans, pigs, and chickens and are occasionally seen in a variety of other species. They typically occur in younger individuals but may be identified at any age in NHPs with reports in infants, juveniles, and adults.^8,19^ The lack of clinical signs produced by the tumor until there is significant renal compromise and reduced handling of NHPs compared with domesticated species is likely responsible for the variable age at diagnosis for NHPs.

Cervical neoplasms have been reported sporadically in rhesus macaques (*M. mulatta*) and baboons (*Papio sp.*), including spontaneous cervical carcinomas in both species, cervical leiomyomas in macaques, and papillomaviral-associated and spontaneous cervical papillomas in baboons.^13,16^ The ETTs are relatively recently described neoplasms in human literature, and the pathogenesis is largely unknown, although the trophoblastic origin is presumed, based on morphologic characteristics and fetal origin supported by the polymerase chain reaction (PCR) analysis. Human chorionic gonadotropin elevations are often supportive of the diagnosis in humans, although this assay was not performed in the case presented here.^9,21,22^ The ETT has been reported previously in NHPs, including in the ovary of a cynomolgus macaque.^6,7,10,26^ Differentiating cervical carcinoma from ETT is notoriously difficult. In human medical literature, ETTs are reported to involve the cervix and may show positive immunoreactivity for cytokeratin, epithelial membrane antigen, inhibin, human placental lactogen, human chorionic gonadotropin, placental alkaline phosphatase, and Mel-cell adhesion molecule.^9,22^ Immunohistochemistry to confirm the presumptive diagnosis of ETT for case 3 were attempted. Cytokeratin immunohistochemistry was not performed in this case as it would not differentiate between cervical carcinoma and ETT. Alpha-inhibin and human chorionic gonadotropin immunohistochemistry was attempted. However, immunoreactivity in owl monkey external and internal control tissues was unreliable making interpretation impossible. Regardless, the histologic appearance of the tumor is consistent with those described in the literature for ETT. Cervical neoplasms are rare in NHPs but may lead to clinical disease; thus, the pelvic region should be part of a thorough necropsy.

Spontaneous neoplasms, particularly in research colonies, can provide unique opportunities to study diseases that affect humans and offer the ability to create models that may improve human health. As such, documenting NHP neoplasms is crucial to add to the body of knowledge of these animals and to improve colony management and species health.

## Supplemental Material

sj-pdf-1-vet-10.1177_03009858241300549 – Supplemental material for Three novel neoplasms in Nancy Ma’s owl monkeys (Aotus nancymaae) Supplemental material, sj-pdf-1-vet-10.1177_03009858241300549 for Three novel neoplasms in Nancy Ma’s owl monkeys (Aotus nancymaae) by Rebecca L Bacon, Carolyn L Hodo, Stephanie J Buchl and Martha E Hensel in Veterinary Pathology
