# Supplementary material for: Three novel neoplasms in Nancy Ma’s owl monkeys (Aotus nancymaae)
Source: Vet Pathol. 2024 Dec 18;62(3):371–5. doi: 10.1177/03009858241300549 (PMC12014950; doi:10.1177/03009858241300549)
Supplement: sj-pdf-1-vet-10.1177_03009858241300549 – Supplemental material for Three novel neoplasms in Nancy Ma’s owl monkeys (Aotus nancymaae) [file sj-pdf-1-vet-10.1177_03009858241300549.pdf]

Supplemental Materials

Three novel neoplasms in Nancy Ma's owl monkeys (*Aotus nancymaae*)

RL Bacon, CL Hodo, ME Hensel

Supplemental Table S1. Details of immunohistochemistry antibodies used in case 1 and attempted use in case 3.

Case	Antibody	Manufacturer	Antibody Clone	Source	Dilution	Positive Control Tissue
1	Pancytokeratin (AE1/AE3)	Biocare Medical[a]	Monoclonal	Mouse	1:100	Rhesus macaque haired skin
1	Vimentin (SP20)	Biocare Medical[a]	Monoclonal	Mouse	1:100	Rhesus macaque tonsil
3	Inhibin alpha (BC/R1)	Biocare Medical[a]	Monoclonal	Mouse	1:100	Rhesus macaque testicle
3	Inhibin alpha	Bioss Antibodies[b]	Polyclonal	Rabbit	1:200	Rhesus macaque testicle
3	hCG	Biocare Medical[a]	Polyclonal	Rabbit	1:200	Human placenta

[a]Biocare Medical, Pacheco, CA, USA.

[b]Bioss Antibodies, Woburn, MA, USA.

Immunohistochemistry Methods

Four micrometer tissue sections were adhered to positively charged slides, deparaffinized, cleared, and brought to water. Slides were immersed in 95˚C Target Retrieval Solution (Dako, Glostrup, Denmark, Europe) for 20 minutes followed by a gradual cooling step. Slides were then immersed and rinsed in wash buffer. The next steps were followed sequentially by a rinse with wash buffer and performed on the Fisher Autostainer: endogenous peroxidase block using dual enzyme block (Dako), Background Sniper (Biocare), Fc Receptor Block (Innovex), primary antibody for 30 minutes, secondary polymer (Envison+, DAKO), 3,3'-diaminobenzidine substrate for 10 minutes (DAKO), and counter stained for 5 minutes with automated hematoxylin (DAKO). Slides were then dehydrated through alcohol gradients and xylene and permanently coverslipped.
